# Pacemaker syndrome due to atrial lead fracture

**DOI:** 10.1002/ccr3.2579

**Published:** 2019-12-05

**Authors:** Thomas Nguyen, Christopher Aldo Rinaldi

**Affiliations:** ^1^ Cardiology Department Guy’s and St Thomas’ NHS Foundation Trust London UK

**Keywords:** lead extraction, lead fracture, pacemaker syndrome, tricuspid regurgitation

## Abstract

Pacemaker syndrome signs and symptoms are shortness of breath, dizziness, fatigue, pulsations in the neck or abdomen, and cannon waves in the neck. Patients with pacemakers experiencing new respiratory or cardiac symptoms should undergo a chest X‐ray and a device interrogation in order to check lead integrity.

## Graphical abstract

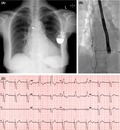

## 1 CASE HISTORY

**Figure 1:**
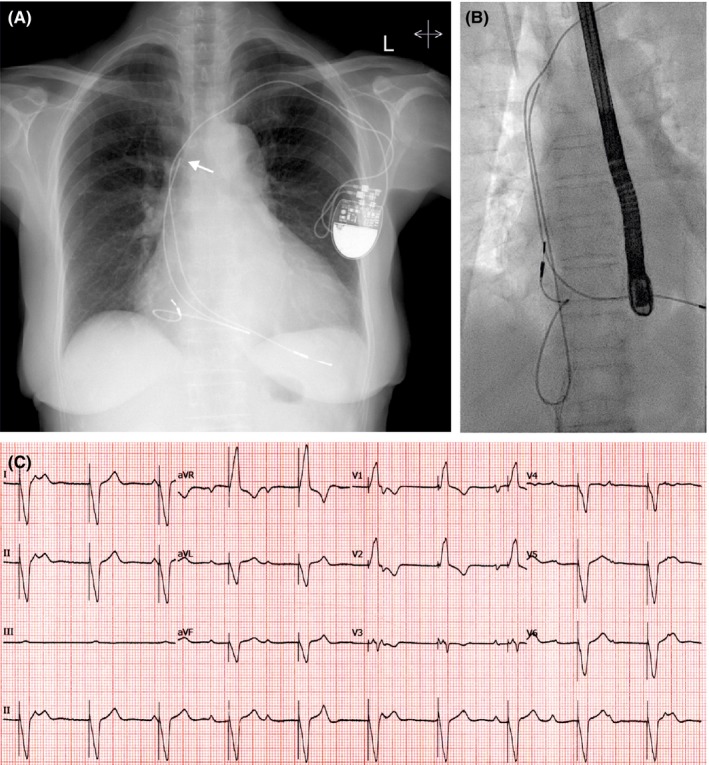
A, Chest X‐ray showing a fracture on the atrial lead (arrow) which loops in the right ventricle. B, Fluoroscopy image during femoral transvenous extraction of the atrial lead. C, 12‐lead ECG showing sinus rhythm and right ventricular pacing without atrial tracking

A 78‐year‐old woman was transferred to our institution for lead extraction. She had a dual‐chamber pacemaker implanted 12 years before for complete heart block. Over the previous months, she developed shortness of breath on exertion and transthoracic echocardiography showed severe tricuspid regurgitation caused by the pacemaker lead. ECG showed loss of atrioventricular synchrony (Figure 1C). Device interrogation demonstrated undersensing and failure to capture on the atrial lead (Medtronic 5092), normal parameters on the right ventricular lead. Chest X‐ray showed an atrial lead fracture with its distal portion looping inside the right ventricle (Figure 1A). Clinical examination was normal except for prominent cannon waves in the neck. Clinical findings were in keeping with pacemaker syndrome caused by the fractured atrial lead. Due to interactions with the atrial lead, the ventricular lead was extracted then the atrial lead was extracted from the femoral route with snares (Figure 1B). An MRI‐compatible dual‐chamber pacemaker was reimplanted. Postoperatively, her shortness of breath improved and she had no more cannon waves.

Pacemaker syndrome is due to the loss of atrioventricular sequential depolarization and contraction.^1^

Complete pacing lead fracture has been reported before but never associated with pacemaker syndrome.^2^

## CONFLICT OF INTEREST

None declared.

## AUTHOR CONTRIBUTIONS

CAR and TN: took part in the care of the patient. TN: wrote the manuscript. CAR: reviewed and supervised the manuscript. All authors approved the final manuscript.
